# Retraction Note to: Excessive mitochondrial fragmentation triggered by erlotinib promotes pancreatic cancer PANC‑1 cell apoptosis via activating the mROS‑HtrA2/Omi pathways

**DOI:** 10.1186/s12935-019-1019-3

**Published:** 2019-11-12

**Authors:** Jun Wan, Jie Cui, Lei Wang, Kunpeng Wu, Xiaoping Hong, Yulin Zou, Shuang Zhao, Hong Ke

**Affiliations:** 1 Department of Pharmacy, Third Clinical Medical College, Three Gorges University, Gezhouba Group Central Hospital, Yichang, 443002 Hubei China; 2 Department of Pathogenic Biology, School of Medicine, China Three Gorges University, Yichang, 443002 Hubei China; 3 Department of Oncology, Third Clinical Medical College, Three Gorges University, Gezhouba Group Central Hospital, No. 60 Qiaohu Lake Road, Xiling District, Yichang, 443002 Hubei China

## Retraction to: Cancer Cell Int (2018) 18:165 10.1186/s12935-018-0665-1

The authors have retracted this article [1] because Figure 4a has been duplicated from Figure 5d in a previously published article [2]. In addition, the article contains sections that substantially overlap with the following articles (amongst others) [3–5]. The data reported in this article are therefore unreliable. Author Hong Ke stated on behalf of all co-authors that they agree to this retraction.
